# High-Quality Draft Genome Sequence of Fischerella thermalis JSC-11, a Siderophilic Cyanobacterium with Bioremediation Potential

**DOI:** 10.1128/mra.00761-22

**Published:** 2022-10-27

**Authors:** Igor Brown, Tanja Woyke, Natalia Ivanova, Nicole Shapiro, Jaime Alcorta, Andrei Chistoserdov, Donald Pan, Svetlana Sarkisova, Susannah G. Tringe

**Affiliations:** a Jacobs Engineering/NASA Johnson Space Center, Houston, Texas, USA; b Department of Energy Joint Genome Institute, Lawrence Berkeley National Laboratory, Berkeley, California, USA; c Department of Molecular Genetics and Microbiology, Biological Sciences Faculty, Pontifical Catholic University of Chile, Santiago, Chile; d Department of Biology, University of Louisiana at Lafayette, Lafayette, Louisiana, USA; e School of Oceanography, Shanghai Jiao Tong University, Shanghai, China; University of Delaware

## Abstract

Here, we report the draft genome sequence of the siderophilic cyanobacterium Fischerella thermalis JSC-11, which was isolated from an iron-depositing hot spring. JSC-11 has bioremediation potential because it is capable of both extracellular absorption and intracellular mineralization of colloidal iron. This genomic information will facilitate the exploration of JSC-11 for bioremediation.

## ANNOUNCEMENT

**FIG 1:**
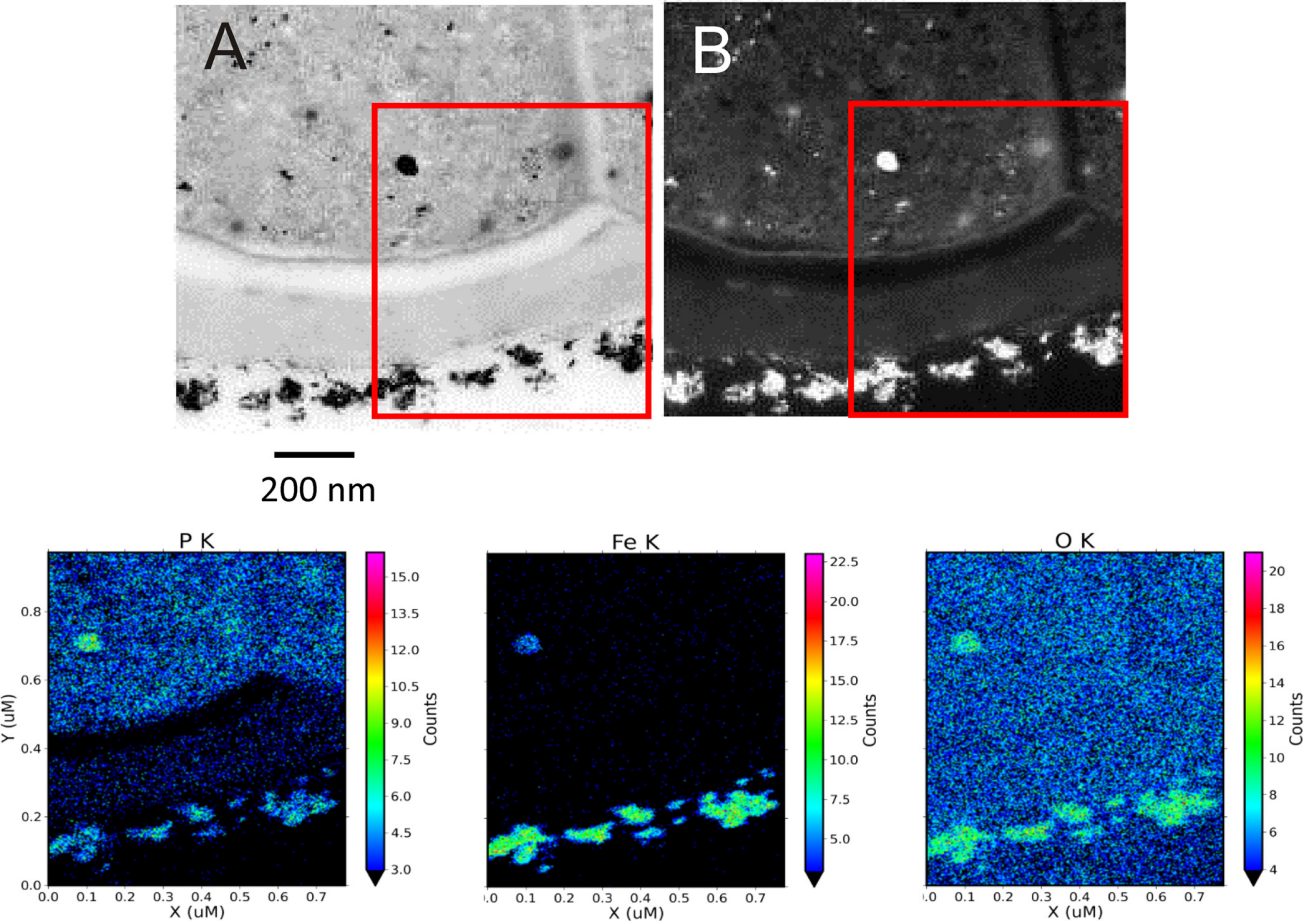
Scanning transmission electron microscopy (STEM) views of extracellular and intracellular iron-rich particles found in JSC-11 cells incubated with 600 μM FeCl_3_·6H_2_O and 0.04 mM Na_2_HPO_4_·7H_2_O. (Upper, left) Bright-field STEM photograph of a JSC-11 cell encrusted with external Fe-bearing precipitates and containing an electron-dense, internal, Fe-rich particle ~100 nm in size. (Upper, right) Dark-field STEM photograph to localize Fe-rich aggregates. (Lower) Quantitative element maps for internal and external particles, showing distribution patterns for phosphorus (P), Fe, and oxygen (O). The electron-dense particles within the JSC-11 cytoplasm contain substantial amounts of P, Fe, and O.

We report the high-quality draft genome sequence of the cyanobacterium JSC-11, which was isolated from an iron-depositing mat from Chocolate Pots Hot Springs (Yellowstone National Park, WY, USA) (temperature of 55°C, pH of 5.6, and total soluble iron concentration of ~20 μM) (1). Single-colony isolation was performed as described previously (2, 3). Because optimal growth occurred at 45 to 55°C and 0.4 to 0.6 mM iron, JSC-11 has been characterized as a thermophilic and siderophilic species (4). Its 16S rRNA sequence (GenBank accession number HM636645.1) shares 100% identity with those of several Fischerella thermalis strains, and it shares their characteristic true-branching morphology (5, 6). Therefore, it was classified as F. thermalis strain JSC-11 (CCMEE 7001). This strain absorbs iron oxides on its exopolymers and produces intracellular iron deposits (Fig. 1), making it a potential candidate for bioremediation (7).

Genomic DNA of JSC-11 was isolated using the UltraClean microbial DNA isolation kit (MoBio Laboratories, USA), following cultivation in DH medium as described previously (8). The draft genome of JSC-11 was generated at the U.S. Department of Energy (DOE) Joint Genome Institute (JGI) using a combination of four next-generation sequencing (NGS) libraries from the same DNA extract. An Illumina GAIIx (9) library was created using the KAPA-Illumina library creation kit (Kapa Biosystems), yielding 31,933,200 reads of 76-bp average length. One 454 Titanium library was created using the GS FLX Titanium rapid library preparation kit (Roche), generating 267,779 reads of 395-bp average length; two paired-end 454 libraries, with average insert sizes of 4 kb and 8 kb, were created according to the method described by Peng et al. (10), generating 602,963 reads of 160- and 188-bp average length, respectively. Kits were used according to the manufacturers’ instructions.

The 454 data were assembled with Newbler v2.3 (9), the Illumina data were assembled with Velvet v1.0.13 (11), and the contigs were computationally shredded into 2-kb and 1.5-kb overlapping fake reads (shreds), which were integrated with the 454 paired-end library reads using parallel Phrap v1.080812 (High Performance Software, LLC). POLISHER (12) was used to correct base errors. Misassemblies were corrected by using Gap Resolution (13), Dupfinisher (14), or sequencing of cloned bridging PCR fragments with subcloning. Gaps were closed manually using Consed (15, 16) and 168 PCR and Bubble PCR primer walks.

Annotation was performed with the Oak Ridge National Laboratory annotation pipeline using Prodigal v1.4 (17), followed by manual curation using the JGI GenePRIMP pipeline (18). The JSC-11 genome is 5,380,000 bp long, with a G+C content of 41.0%, and has 99.76% completeness and 0.0% contamination according to CheckM v1.1.3 (19). It contains 4,367 coding genes, 1 rRNA operon, and 43 tRNA genes.

The JSC-11 genome may shed light on specific mechanisms of iron homeostasis in siderophilic cyanobacteria, because it encodes proteins predicted to be involved in iron transport (21 total) or maintaining a balance between intracellular Fe^2+^ and Fe^3+^ (5 total). Genomic analysis also revealed that JSC-11 may be capable of far-red-light photoacclimation (20).

### Data availability.

The GenBank accession number for the 16S rRNA gene is HM636645.1. The GenBank assembly accession number is GCA_000231365.2. The NCBI RefSeq assembly accession number is GCF_000231365.1. The BioProject accession number is PRJNA61093. The nucleotide GenBank accession number is AGIZ00000000.1. The SRA accession numbers for the raw sequences are SRR21655585, SRR21655586, SRR21655587, and SRR3927276.

## References

[1] Fortney NW, He S, Converse BJ, Boyd ES, Roden EE. 2018. Investigating the composition and metabolic potential of microbial communities in Chocolate Pots hot springs. Front Microbiol 9:2075. doi:10.3389/fmicb.2018.02075. 30245673 PMC6137239

[2] Brown II, Mummey D, Cooksey KE. 2005. A novel cyanobacterium exhibiting an elevated tolerance for iron. FEMS Microbiol Ecol 52:307–314. doi:10.1016/j.femsec.2004.11.020. 16329916

[3] Brown II, Bryant DA, Casamatta D, Thomas-Keprta KL, Sarkisova SA, Shen G, Graham JE, Boyd ES, Peters JW, Garrison DH, McKay DS. 2010. Polyphasic characterization of a thermotolerant siderophilic filamentous cyanobacterium that produces intracellular iron deposits. Appl Environ Microbiol 76:6664–6672. doi:10.1128/AEM.00662-10. 20709851 PMC2950469

[4] Brown I, Allen C, Mummey DL, Sarkisova S, McKay DS. 2007. Iron-tolerant cyanobacteria, p 425–442. *In* Seckbach J (ed), Algae and cyanobacteria in extreme environments. Springer, Dordrecht, Netherlands. doi:10.1007/978-1-4020-6112-7_23.

[5] Alcorta J, Vergara-Barros P, Antonaru LA, Alcamán-Arias ME, Nürnberg DJ, Díez B. 2019. *Fischerella thermalis*: a model organism to study thermophilic diazotrophy, photosynthesis and multicellularity in cyanobacteria. Extremophiles 23:635–647. doi:10.1007/s00792-019-01125-4. 31512055

[6] Rippka R, Deruelles J, Waterbury JB, Herdman M, Stanier RY. 1979. Generic assignments, strain histories and properties of pure cultures of cyanobacteria. Microbiology 111:1–61. doi:10.1099/00221287-111-1-1.

[7] Al-Amin A, Parvin F, Chakraborty J, Kim Y-I. 2021. Cyanobacteria mediated heavy metal removal: a review on mechanism, biosynthesis, and removal capability. Environ Technol Rev 10:44–57. doi:10.1080/21622515.2020.1869323.

[8] Brown I, Tringe SG, Ivanova N, Goodwin L, Shapiro N, Alcorta J, Pan D, Chistoserdov A, Sarkisova S, Woyke T. 2021. High-quality draft genome sequence of the siderophilic and thermophilic *Leptolyngbyaceae* cyanobacterium JSC-12. Microbiol Resour Announc 10:e00495-21. doi:10.1128/MRA.00495-21. PMC8223812 34165332

[9] Margulies M, Egholm M, Altman WE, Attiya S, Bader JS, Bemben LA, Berka J, Braverman MS, Chen Y-J, Chen Z, Dewell SB, Du L, Fierro JM, Gomes XV, Godwin BC, He W, Helgesen SHC, Irzyk GP, Jando SC, Alenquer MLI, Jarvie TP, Jirage KB, Kim J-B, Knight JR, Lanza JR, Leamon JH, Lefkowitz SM, Lei M, Li J, Lohman KL, Lu H, Makhijani VB, McDade KE, McKenna MP, Myers EW, Nickerson E, Nobile JR, Plant R, Puc BP, Ronan MT, Roth GT, Sarkis GJ, Simons JF, Simpson JW, Srinivasan M, Tartaro KR, Tomasz A, Vogt KA, Volkmer GA, Wang SH, Wang Y, Weiner MP, Yu P, Begley RF, Rothberg JM. 2005. Genome sequencing in microfabricated high-density picolitre reactors. Nature 437:376–380. doi:10.1038/nature03959. 16056220 PMC1464427

[10] Peng Z, Zhao Z, Nath N, Froula JL, Clum A, Zhang T, Cheng J-F, Copeland AC, Pennacchio LA, Chen F. 2012. Generation of long insert pairs using a Cre-LoxP inverse PCR approach. PLoS One 7:e29437. doi:10.1371/journal.pone.0029437. 22253722 PMC3253782

[11] Zerbino DR, Birney E. 2008. Velvet: algorithms for de novo short read assembly using de Bruijn graphs. Genome Res 18:821–829. doi:10.1101/gr.074492.107. 18349386 PMC2336801

[12] LaButti K, Foster B, Lowry S, Trong S, Goltsman E, Lapidus A. 2008. POLISHER: a tool for using ultra short reads in microbial genome finishing. LBNL report LBNL-639E. https://escholarship.org/uc/item/9wh0d388.

[13] Labutti K, Foster B, Lapidus A. 2017. Gap resolution. https://www.osti.gov/doecode/biblio/57346.

[14] Han C, Chain P. 2006. Finishing repeat regions automatically with Dupfinisher, p 141–146. *In* Arabina HR, Valafar H (ed), Proceeding of the 2006 International Conference on Bioinformatics & Computational Biology, BIOCOMP 06, Las Vegas, Nevada, USA, June 26–29, 2006. https://researchr.org/publication/biocomp%3A2006.

[15] Ewing B, Green P. 1998. Base-calling of automated sequencer traces using Phred. II. Error probabilities. Genome Res 8:186–194. doi:10.1101/gr.8.3.186. 9521922

[16] Ewing B, Hillier L, Wendl MC, Green P. 1998. Base-calling of automated sequencer traces using Phred. I. Accuracy assessment. Genome Res 8:175–185. doi:10.1101/gr.8.3.175. 9521921

[17] Hyatt D, Chen G-L, LoCascio PF, Land ML, Larimer FW, Hauser LJ. 2010. Prodigal: prokaryotic gene recognition and translation initiation site identification. BMC Bioinformatics 11:119. doi:10.1186/1471-2105-11-119. 20211023 PMC2848648

[18] Pati A, Ivanova NN, Mikhailova N, Ovchinnikova G, Hooper SD, Lykidis A, Kyrpides NC. 2010. GenePRIMP: a gene prediction improvement pipeline for prokaryotic genomes. Nat Methods 7:455–457. doi:10.1038/nmeth.1457. 20436475

[19] Parks DH, Imelfort M, Skennerton CT, Hugenholtz P, Tyson GW. 2015. CheckM: assessing the quality of microbial genomes recovered from isolates, single cells, and metagenomes. Genome Res 25:1043–1055. doi:10.1101/gr.186072.114. 25977477 PMC4484387

[20] Gan F, Bryant DA. 2015. Adaptive and acclimative responses of cyanobacteria to far-red light: responses of cyanobacteria to far-red light. Environ Microbiol 17:3450–3465. doi:10.1111/1462-2920.12992. 26234306

